# Positive selection and functional divergence of farnesyl pyrophosphate synthase genes in plants

**DOI:** 10.1186/s12867-017-0081-4

**Published:** 2017-02-04

**Authors:** Jieying Qian, Yong Liu, Naixia Chao, Chengtong Ma, Qicong Chen, Jian Sun, Yaosheng Wu

**Affiliations:** 1 Key Laboratory of Biological Molecular Medicine Research of Guangxi Higher Education, Department of Biochemistry and Molecular Biology, Guangxi Medical University, Nanning, Guangxi People’s Republic of China; 2 Schools of Pharmacy, Guangdong Medical University, Dongguan, Guangdong People’s Republic of China

**Keywords:** Biological evolution, Farnesyl pyrophosphate synthase, Positive selection, Terpenoid biosynthesis

## Abstract

**Background:**

Farnesyl pyrophosphate synthase (FPS) belongs to the short-chain prenyltransferase family, and it performs a conserved and essential role in the terpenoid biosynthesis pathway. However, its classification, evolutionary history, and the forces driving the evolution of FPS genes in plants remain poorly understood.

**Results:**

Phylogeny and positive selection analysis was used to identify the evolutionary forces that led to the functional divergence of FPS in plants, and recombinant detection was undertaken using the Genetic Algorithm for Recombination Detection (GARD) method. The dataset included 68 FPS variation pattern sequences (2 gymnosperms, 10 monocotyledons, 54 dicotyledons, and 2 outgroups). This study revealed that the FPS gene was under positive selection in plants. No recombinant within the FPS gene was found. Therefore, it was inferred that the positive selection of FPS had not been influenced by a recombinant episode. The positively selected sites were mainly located in the catalytic center and functional areas, which indicated that the 98S and 234D were important positively selected sites for plant FPS in the terpenoid biosynthesis pathway. They were located in the FPS conserved domain of the catalytic site. We inferred that the diversification of FPS genes was associated with functional divergence and could be driven by positive selection.

**Conclusions:**

It was clear that protein sequence evolution via positive selection was able to drive adaptive diversification in plant FPS proteins. This study provides information on the classification and positive selection of plant FPS genes, and the results could be useful for further research on the regulation of triterpenoid biosynthesis.

**Electronic supplementary material:**

The online version of this article (doi:10.1186/s12867-017-0081-4) contains supplementary material, which is available to authorized users.

## Background

Triterpenoids are a large class of plant secondary metabolites. They enable plants to withstand pathogens and pests [1, 2]. Many different plant species synthesize triterpenoid saponins during normal growth and development [3]. In clinical medicine, it has been shown that triterpene saponins have anti-tumor, anti-inflammatory, and anti-viral activities. They also help lower cholesterol and elevate immunity [4–11]. Generally, the biosynthetic pathway for terpenoids can be divided into four or five stages. These are the formation of IPP (isopentenyl diphosphate, C5 unit), GPP (geranyl diphosphate, C10 unit), FPP (farnesyl diphosphate, C15 unit), squalene (C30 unit), 2, 3-oxidosqualene, and triterpenoid [3, 12, 13]. Farnesyl pyrophosphate synthase (FPS) catalyzes FPP formation. FPS has been widely found in lower green algae up to higher eudicot plants and has been cloned from various plants [14–22]. However, its origin, evolution, and structural and functional divergence remain poorly understood.

Farnesyl pyrophosphate synthase belongs to the short-chain prenyltransferase family [23] and it accelerates the head-to-tail condensation reaction of dimethylallyl pyrophosphate (DMAPP) with two molecules of isopentenyl pyrophosphate (IPP) to form FPP [24], which is the precursor of all sesquiterpenes and triterpenoids [25]. FPS provides substrate FPP to squalene synthase and sesquiterpene synthase [15]. Squalene synthase plays a role in steroid and triterpenoid synthesis, which are involved in cell membrane system building. Sesquiterpene synthase plays a role in the synthesis of cyclic sesquiterpene compounds [26]. FPS mainly affects sesquiterpene compounds [22] and then squalene synthase (SS) primarily controls downstream triterpenoid synthesis [27–29]. The large FPS functional diversity suggests that it may be subject to positive Darwinian selection. The conserved domains 90–104 (LVLDDIMDSSHTRRG) and 225–237 (MGTYFQVQDDYLD) of *Panax notoginseng* FPS (*PnFPS*) have important effects on the catalytic activity of isopentenyl pyrophosphate synthase (Trans-IPPS) in downstream products [30]. However, it is not known how the FPS genes evolved and functionally diverged, or whether positive selection is associated with the two important functional domains. Furthermore, it remains unclear what the evolutionary relationships are between some essential catalytic sites. In this study, we analyzed nucleotide and amino acid residue divergence in the FPS genes from 68 species of land plants. Likelihood methods that utilized the site-model, branch-model, and branch-site model were used to investigate potential positive selection patterns for plant FPS.

## Results

### Origins of the FPS genes during plant evolution

**Fig. 1:**
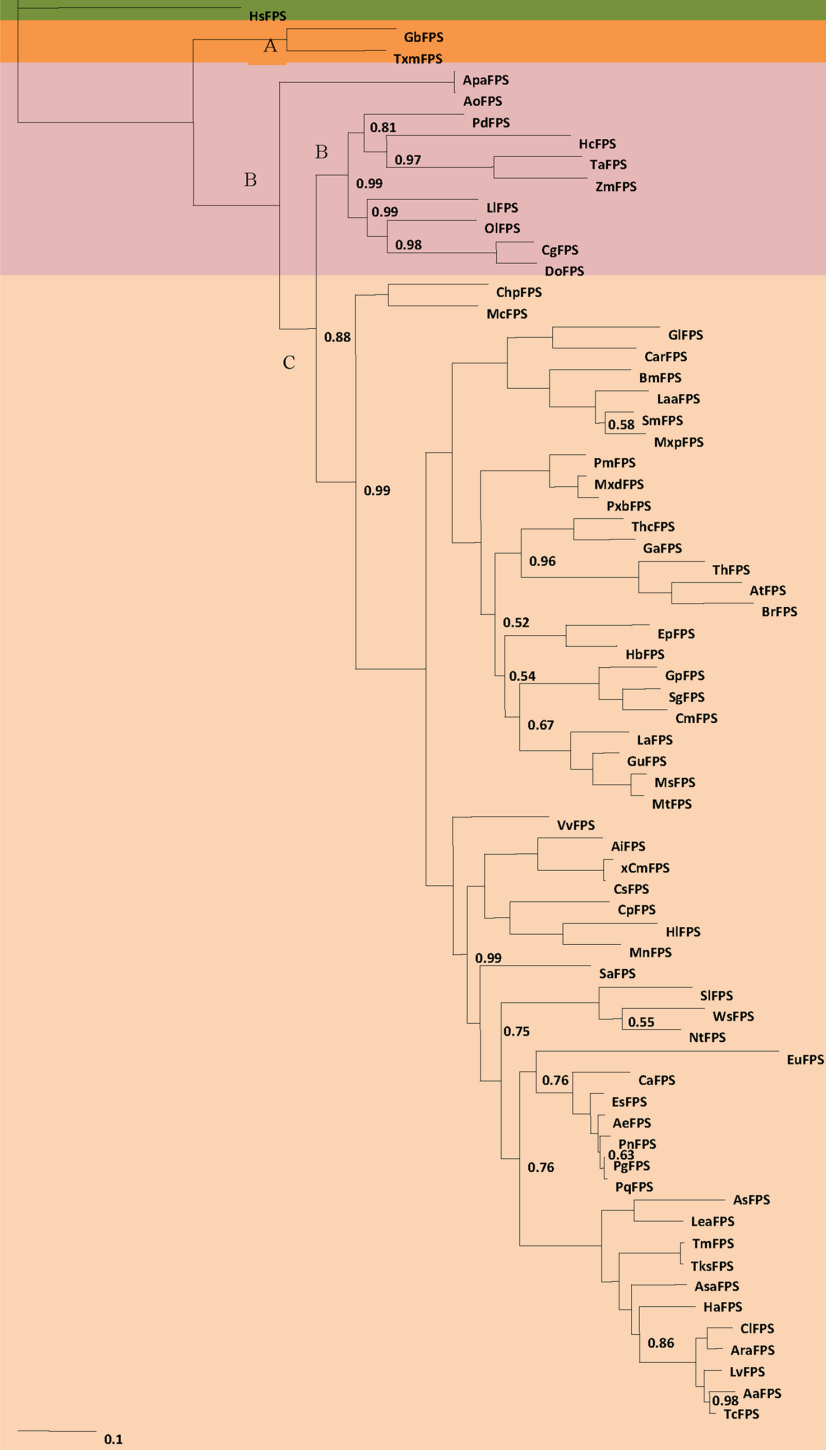
Phylogenetic tree of terrestrial plant FPS. The phylogenetic tree of plant FPSs was constructed through the Bayesian analyses. Posterior probabilities are labeled above branches. *Chlamydomonas reinhardtii (CrFPS)* and *Huperzia serrate (HsFPS)* were used as outgroups. The clades of gymnosperms, monocotyledons and dicotyledons were labeled as *A*, *B* and *C*, respectively. The *numbers* indicate the Bayesian probabilities for each phylogenetic clade. Posterior probability values were to only show the pp values smaller than 1.0 with the tree

A rooted maximum-likelihood (ML) phylogenetic tree based on codon alignment was produced by the Bayesian method in order to explore the origin and evolutionary history of FPS genes among plants. The FPS cDNA sequences from 68 species were used to reconstruct a phylogenetic tree. In addition, we used the Bayesian posterior probability (PP) to evaluate all clade supports. The analysis revealed that the FPS genes mainly fell into one of three general groups: gymnosperms (A), monocotyledons (B), and dicotyledons (C) (Fig. 1). The monocotyledons FPS isoforms are a highly supported monophyletic group and are thus separated from the dicot isoforms. The dicotyledons group contains representatives from all of the available dicots, including verified FPS sequences from *Panax notoginseng*, *Panax ginseng*, *Gynostemma pentaphyllum*, etc. The gymnosperm FPS also formed a separate cluster that was closest to the monocots. The phylogeny showed that FPS genes consist of several distinct branch clusters, indicating that the formation of the paralogous lineages occurred before divergence of the individual species [31], and that *Chlamydomonas reinhardtii (CrFPS)* and *Huperzia serrate (HsFPS)* were outgroups of the assigned lineages. In plants, gene evolution leading to functional divergence plays a crucial role in the diversification of biochemical metabolites [32]. These findings were consistent with previous studies on the phylogenetic classification of terrestrial plants. Thus, the terrestrial plant phylogenetic tree for FPS genes may reflect the genetic relationships among different species. Based on the lineages of the tree, we inferred that the metabolites produced by different species varied as the accompanying metabolic pathway diverged. Plant FPS is located at a branch point of the terpenoid synthesis pathway and is responsible for directing carbon flow away from the central portion of the isoprenoid pathway [30]. Two types of terpenoids occurred. These were tetracyclic and pentacyclic triterpenoids. For example, ginsenoside, the main component of ginseng, is a dammarane tetracyclic triterpenoid. The oleanane-type pentacyclic triterpenoids are the most widespread, and hitherto most extensively studied compounds in the family Araliaceae, family Cucurbitaceae, and family Leguminosae.

### Detection of recombinant episodes

We were able to detect positive selection pressures using the evolutionary phylogenetic tree. However, recombination can have a profound impact on the evolutionary process [33] and can adversely affect the power and accuracy of phylogenetic reconstruction, molecular clock inference, and the detection of positively-selected sites [34–36]. Therefore, the recombination factor must be considered before performing positive selection analysis. In our study, Mafft software was used to align the 68 FPS sequences and convert the format to fasta. The aligned sequences file was used by the Genetic Algorithm for Recombination Detection (GARD) and Recombination Detection Program (RDP) methods to detect the recombinant events. The GARD and RDP analysis found no recombinant within the FPS genes. Therefore, it was inferred that the positive selection of FPS has not been influenced by a recombinant episode.

### Positively selected sites in the FPS family and their putative biological significance

The site-specific model, the branch model, the branch-site model, and PAML package version 4.4 were used to detect the selective pressure on the FPS family in plants. After removing the gaps, all the amino acids sites were analyzed using the CodeML program. In the site model, none of the positive selection sites was detected by the M0 vs. M3 or M2a vs. M1a model. However, the alternative models, M3 and M8, may fit the data significantly better than the null models, M0 and M7 (for M3 vs. M0, 2ΔL = 2715.02, p < 0.001; for M8 vs. M7, 2ΔL = 9346.66, p < 0.001), but only M8 identified several sites with an ω value significantly greater than 1. Therefore, at the PP > 95% level, 39 amino acid sites were identified as being under positive selection by M8 (Table 1), including 28 positive selection sites with a PP > 99% (Table 1) and 11 sites as potential targets of positive selection with a PP > 0.95 (1M, 2S, 6T, 10E, 29D, 111L, 125L, 176S, 195S, 310K, and 326A). Positive selection may only happen during specific stages of evolution or in specific branches, which means that positive selection may only affect some branches. Therefore, we used a branch-specific model to detect positive selection. The branch model suggested that the free ratio model was significantly higher than the one ratio model (2ΔlnL = 256.64, p = 0.00), which indicated that there was heterogeneous selection among branches. The selective pressure on the different branches and sites was investigated by using the branch-site model to directly search for the positively-selected amino acid sites. Branch-site model was used to search for amino acid sites that underwent positive selection in branches a, b, and c, and then fixed the three branches as foreground branches in the branch site model. According to the likelihood ratio test (LRT) for the branch-site (Table 1), comparisons of BSa1 vs. BSa0-fix (2ΔlnL = 10.56, p = 0.0012), BSb1 vs. BSb0-fix (2ΔlnL = 10.12, p = 0.01), and BSc1 vs. BSc0-fix (2ΔlnL = 9.98, p = 0.01), were significantly different. Naive Empirical Bayes (NEB) analysis and Bayes Empirical Bayes (BEB) analysis were undertaken, but the BEB analysis showed the posteriori probability of the positive selection sites better than the NEB analysis. The positive pressure computation showed that there were three amino acid sites (98S, 148D, 234D) in the branch with a p < 0.01 for BSa1 vs. BSa0-fix, which were considered to have undergone positive selection. The analysis showed that (1) FPS genes suffered from positive selection during the plant evolutionary process; and (2) some representative positively-selected sites were located in the catalytic region. These features suggested that positive selection sites located in the functional domain of FPS are important components of the FPS functional structure.

**Table 1:** Positive selection sites of FPS tested through the site model, branch model and branch-site model

Model	Estimate of parameters	lnL	LRT pairs	df	2ΔlnL	*P* value	Positive selection sites
*Site model*							
M0:one ratio	ω = 0.11676	−30867.39					
M3:discrete	p0 = 0.49134, p1 = 0.296497, p2 = 0.21217, ω0 = 0.01740, ω1 = 0.15430, ω2 = 0.50703	−29509.88	M0/M3	3	2715.02	0	none
M1a:neutral	p0 = 0.75883, p1 = 0.24117, ω0 = 0.07661, ω1 = 1.00000	−30122.51	M1a/M2a	2	0	1	none
M2a:selection	p0 = 0.75883, p1 = 0.15391, p2 = 0.08726, ω0 = 0.07661, ω1 = 1.0000, ω2 = 1.0000	−30122.51					
M7:beta	p = 0.37262, q = 1.74173	−29440.75					
M8:beta&ω	p0 = 1.0000, p = 0.28555, q = 1.02636, (p1 = 0.00000), ω2 = 2.36785	−34114.08	M7/M8	2	9346.66	0	3D, 7R, 14V, 21N, 25F, 27F, 34W, 47K, 59K, 60L, 65K, 98S, 99S, 181P, 207S, 213K, 233D, 293E, 275F, 286A, 251D, 252I, 270E, 302D, 305A, 309S, 336G, 342Q (all were^a^)
*Branch model*							
Model 0:(one-ratio)	ω = 0.11676	−30867.39	M0/Free model	135	258.64	0	none
Free model	ωa = 1.0131, ωb = 1.3249, ωc = 540.6926	−30738.07					
*Branch*-*site model*							
BSa1	p0 = 0.00006, p1 = 0.00001, p2a = 0.85238, p2b = 0.14755, b:ω0 = 0.07506, ω1 = 1.00000, ω2a = 0.07506, ω2b = 1.00000, f:ω0 = 0.07506, ω1 = 1.00000, ω2a = 1.00000, ω2b = 1.00000	−25664.61	BSa1/BSa0-fix	1	10.56	0	98S, 148D, 234D
BSa0_fix	p0 = 0.81966, p1 = 0.14204, p2a = 0.03264, p2b = 0.00566, b:ω0 = 0.07521, ω1 = 1.00000, ω2a = 0.07521, ω2b = 1.00000, f:ω0 = 0.07521, ω1 = 1.00000, ω2a = 1.00000, ω2b = 1.00000	−25669.89					none
BSb1	p0 = 0.02224, p1 = 0.00386, p2a = 0.82997, p2b = 0.14393, b:ω0 = 0.07528, ω1 = 1.00000, ω2a = 0.07528, ω2b = 1.00000,f:ω0 = 0.07528, ω1 = 1.00000, ω2a = 1.00000, ω2b = 1.00000	−25663.83	BSb1/BSb0-fix	1	10.12	0	none
BSb0_fix	p0 = 0.10932, p1 = 0.01896, p2a = 0.74286, p2b = 0.12886, b:ω0 = 0.07528, ω1 = 1.00000, ω2a = 0.07528, ω2b = 1.00000, f:ω0 = 0.07528, ω1 = 1.00000, ω2a = 8.15518, ω2b = 8.15518, f:ω0 = 0.07528, ω1 = 1.00000, ω2a = 8.15518, ω2b = 8.15518	−25668.89					
BSc1	p0 = 0.03778, p1 = 0.00655, p2a = 0.81443, p2b = 0.14124, b:ω0 = 0.07526,ω1 = 1.00000,ω2a = 0.07526,ω2b = 1.00000, f:ω0 = 0.07526, ω1 = 1.00000, ω2a = 1.00000, ω2b = 8.15518	−25664.75	BSc1/BSc0-fix	1	9.98	0	none
Sc0_fix	p0 = 0.01751, p1 = 0.00303, p2a = 0.83486, p2b = 0.14461, b:ω0 = 0.07526, ω1 = 1.00000, ω2a = 0.07526, ω2b = 1.00000, f:ω0 = 0.07526, ω1 = 1.00000, ω2a = 1.00000, ω2b = 1.00000	−25669.74					

Selection analysis by site model was performed using CodeML implemented in PAML. Significant tests at 1% cut off

*lnL* log-likelihood values, *LRT* likelihood ratio test, *ω2* average dN/dS ratio for sites subject to positive selection, *p and q* shape parameters for the beta distribution of ω, *p0, p1, and p2* proportions of codons subject to purifying selection, neutral evolution, and positive selection, respectively, *df* degrees of freedom, *2ΔlnL* twice the log-likelihood difference of the model compared

^a^ Posterior probability >99%

### Protein structural characteristics of FPS in plants

**Fig. 2:**
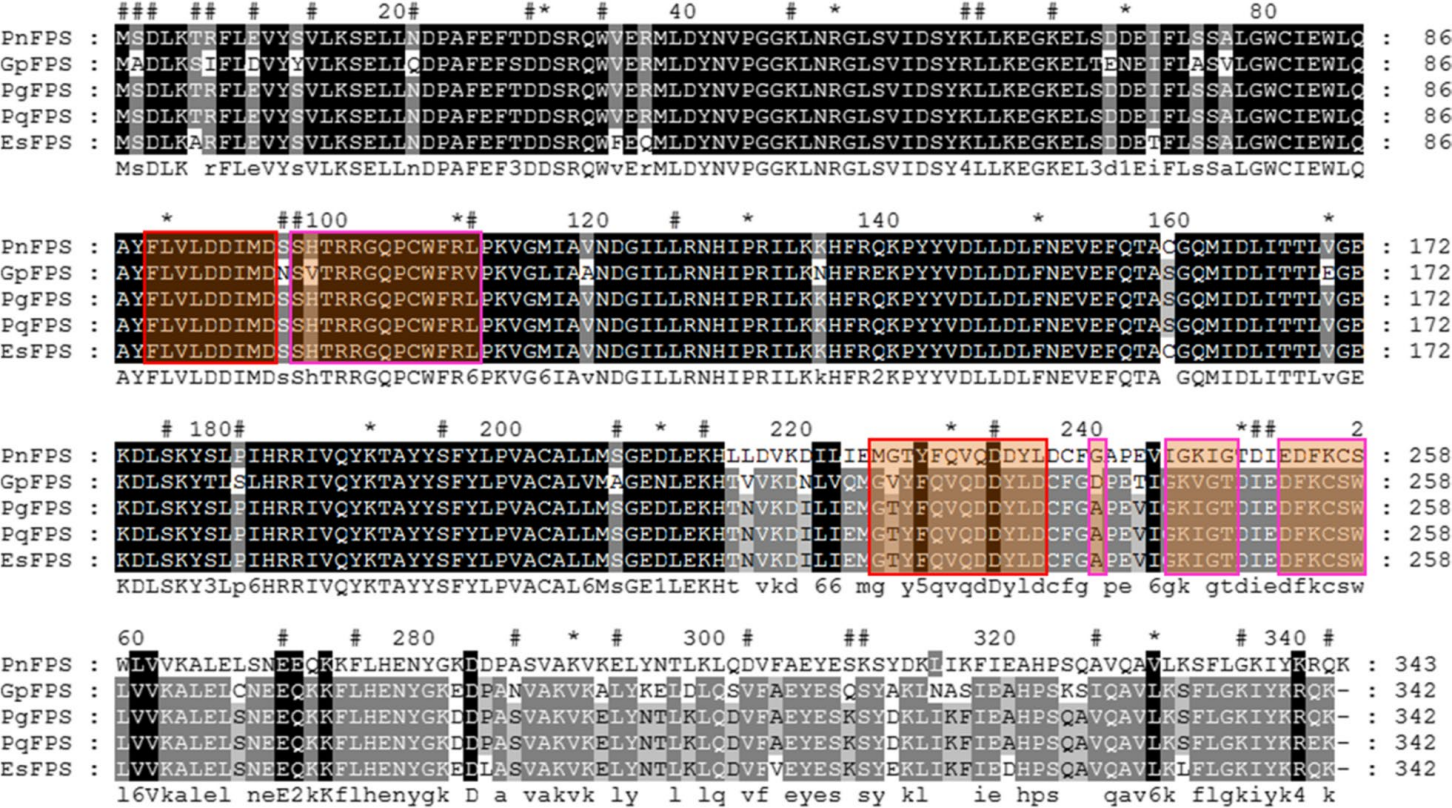
Multi-alignment of the amino acid sequences of partial terrestrial plant FPS. *PnFPS*, *PgFPS*, *EsFPS*, *PqFPS*, and *GpFPS* represent farnesyl pyrophosphate synthase cloned from *Panax notoginseng, Panax ginseng, Eleutherococcus senticosus, Panax quinquefolium*, and *Gynostemma pentaphyllum*, respectively. The positive selection sites for *FPS* in the above five common medicinal plants were marked and displayed through GeneDoc (http://www.nrbsc.org/gfx/genedoc). *PnFPS* was used as the reference sequence. The conserved sites were shaded. *Hash symbol* positive selection site; *red box* conserved sites of trans-isoprenyl diphosphate synthases (Trans IPPS); *carmine box* active site lid residues

In addition to the above-mentioned phylogenetic and the positive selection FPS analysis, we also conducted detailed structural studies based on the two-dimensional model containing the protein sequence alignment of the FPS in several important medicinal herbs, such as *Panax ginseng* (*PgFPS*), *Panax quinquefolium* (*PqFPS*), *Gynostemma pentaphyllum* (*GpFPS*), *Panax notoginseng* (*PnFPS*), and *Eleutherococcus senticosus* (*EsFPS*). *PnFPS* was used as the reference sequence. These FPSs shared a high level of sequence similarity in the coding region. The structure of the FPS members is highly conserved. The conserved sites (shaded) and the functional areas are shown in Fig. 2. The observations suggested that these areas may undergo positive Darwinian selection or an increase in the fixation of neutral mutations due to the relaxation of functional constraints. We mapped these sites onto the model as well as their sequence alignments. The results showed that the distribution of these sites was largely disordered, but a few sites were concentrated in some special FPS spatial locations.

### Distributions of possible positive selection sites on FPS three dimensional structures

**Fig. 3:**
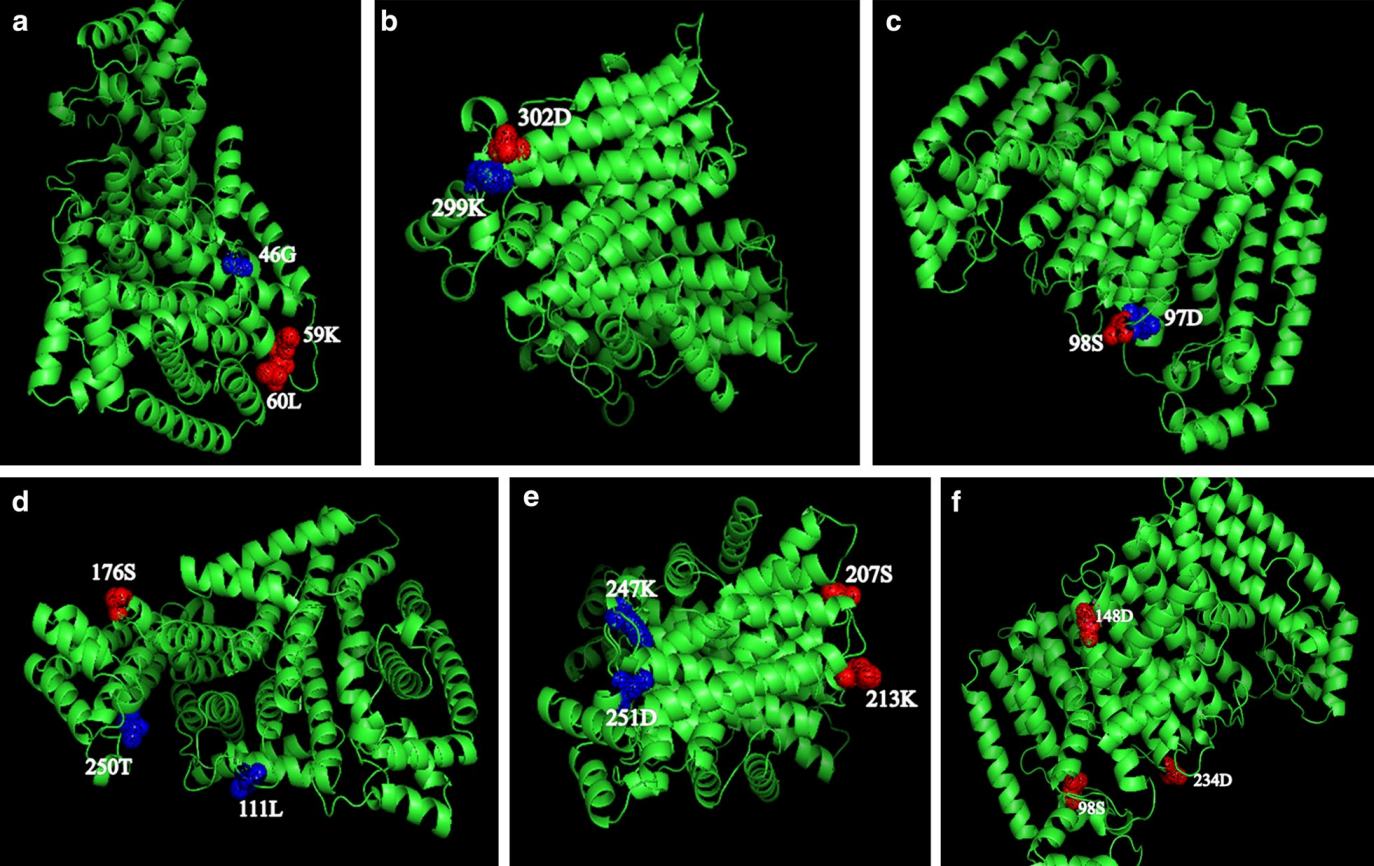
Positive selection sites (*red*) and functional sites (*blue*) displaying on the FPS 3D structure by PYMOL software version 1.5. **a** Involved in the N-myristoylation site; **b** involved in the protein kinase C phosphorylation site; **c** involved in chemical binding site; **d** involved in active site lid residues; **e** positive selection sites identified by the branch-site model; **f** involved in substrate-Mg^2+^ binding site

We predicted the positive selection sites using the BEB method. Thirty-nine sites were identified as positively selected at a BEB posterior probability threshold of 95% in the site-model. In order to draw positive selected sites onto a plant FPS three-dimensional model, we first built an energy-minimized model using a homology modeling approach [37]. We took the protein structure of *Panax notoginseng* as an example and analyzed the relationship between positive selection sites and functional sites. The PDB data was produced in Swiss model (http://swissmodel.expasy.org/), where the highest sequence similarity identified in the PSI-BLAST analysis corresponded to the FPS. We mapped three positively selected sites (98S, 148D, and 234D) and tested them in the branch-site model. Other important positively selected sites tested in the site model were mapped onto the surface of the three-dimensional structure by Pymol (http://PyMOLwiki.org). As shown in Fig. 3, positively-selected 59K and 60L were relatively adjacent to the acylated 46G site in the spatial structure (Fig. 3a: involved in N-myristoylation site 46G), and 302D was near to the protein kinase C phosphorylation site in the spatial structure (Fig. 3b: Involved in the protein kinase C phosphorylation site). In Fig. 3c, positively selected site 98S was close to the chemical binding site 97D. Furthermore, in the 111L and 250T active sites, positively-selected site 176S was significantly related to the active sites (Fig. 3d: involved in active site lid residues 111L and 250T). In the highly conserved domain, positive selection sites 98S, and 234D were located in the important DDXX (XX)D aspartate-rich domains (Fig. 3e: positive selection sites tests in the branch-site model). Positive selection sites 207S and 213K were close to the substrate-Mg^2+^ binding sites 247K and 251D (Fig. 3f: involved in substrate-Mg^2+^ binding site 247K and 251D). All of these positively-selected sites may be key amino acids for this important functional region.

## Discussion

FPS plays a vital role in the isoprenoid biosynthesis pathway. The reaction catalyzed by FPS is considered the rate-limiting step and determines the flow fate of farnesyl diphosphate [15, 22, 30]. In this study, we reported the molecular evolution of positive selection sites in plant FPS genes for the first time. The gene expression analysis showed that FPS genes could increase terpenoid accumulation in plants [15, 38, 39]. In our study, we combined molecular phylogenetic analysis, putative biological significance, and protein structure analysis to clarify the evolutionary mechanisms. However, how FPS improves the triterpenoid content in the biosynthesis pathway is still not clear, and their biological roles in many species are also poorly understood.

As the number of FPS gene sequences cloned in our laboratory and collected from the database increased, it became more feasible to explore the evolutionary relationships and the functional diversity of the FPS family. In this study, 68 sequences were used for phylogenetic reconstruction by Bayesian methods. The phylogenetic analysis showed that FPS gene formation occurred before the divergence of individual species. The phylogenetic tree allowed us to investigate FPS evolution and to further understand the relationship between FPS structure and function in plants. These results are consistent with the phylogenetic classification of terrestrial plants and similar to the functional divergence analysis. The phylogenetic analysis clearly showed how FPS was classified, which may affect its functional divergence.

Positive selection is the retention and spread of advantageous mutations throughout a population and has long been considered synonymous with protein functional shifts [40]. Previous research found that positively-selected genes are more likely to interact with each other than genes not under positive selection [41].

In the evolutionary history of many microorganisms, positive selection and homologous recombination are two indispensable forces that drive adaptation to new niches. Therefore, before undertaking the positive selection analysis, we detected potential recombination events in order to assure the accuracy of any positive selections found. GARD found no evidence of recombination, which meant that the positive selections detected were statistically reliable. The selection events on coding sequences could affect gene expression regulation. Therefore, it is vital to detect positively-selected sites on the FPS ORF in order to get a further insight into the relationship between its structure and function. Site model, branch model, and branch-site model were used to detect positive selection among pre-specified groups. The ω values from the site model analysis did not fit the data well enough to describe the variability under selection pressure across amino acid sites. However, the branch model results showed that the ω ratios varied among clades, which meant that this model could be used to evaluate some sites in specific clades of the FPS phylogenic tree. Using molecular adaptive evolution and the positive selection principle to search corresponding functional sites can provide valuable reference information for FPSs that influence the regulation of synthetic triterpenoids. About 20 years ago, several structural FPS genes from *Homo sapiens*, *Rattus rattus, Callus gallus, Saccharomyces cerevisiae, Escherichia coli,* and *Bacillus stearothermophilus* were identified and characterized, and five regions with highly conserved residues and sequence comparisons revealed two conserved DDXX(XX)D aspartate-rich domains [42], which were considered to be binding sites for the diphosphate moieties in IPP and allylic substrates. Now, many plant FPS genes have been cloned and identified too [14, 18, 19, 43, 44]. As shown in the space structure of *PnFPS* in Fig. 2, the positively-selected 59K site is overlapped in protein kinase C phosphorylation sites and 207S coincides with casein kinase II phosphorylation sites. Positions 90–104 (LVLDDIMDSSHTRRG) and 225–237 (MGTYFQVQDDYLD) in *PnFPS* contain the isopentenyl pyrophosphate synthase (Trans-IPPS) conserved domain of the catalytic site, and positive selection sites 98S and 99S are in the conserved domains. The first aspartate-rich region is an FPS chain length determination (CLD) region for the consecutive condensations of isopentenyl diphosphate with allylic diphosphates. A conversion analysis of archaeal geranylgeranyl pyrophosphate synthase (GGPS**)** to FPS inferred that the archaeal GGPSs had evolved into type I and type II FPSs in eukaryotes and prokaryotes, respectively, and that the conserved CLD region made significant differences to some important FPS functions [45]. It was predicted that the region around the first aspartate-rich motif was essential for the product specificity of all FPP synthases and that the aromatic amino acid on the fifth amino acid before the first aspartate-rich motif (DDXX (XX)D, FARM) had been replaced. In this study, the positive selection sites 98S and 99S in plant FPS were found to be close to the first conserved motif (DDIMD). Therefore, 98S and 99S might be important sites that affect the biochemical function of plant FPS. Moreover, the site 59K coincided with protein kinase C phosphorylation, which indicated that 59K might undergo positive selection, so we inferred that this site could be related to protein tyrosine phosphorylation. A mutation in this site might change the downstream reactions during secondary metabolite biosynthesis. 207S also underwent a positive pressure that corresponded to the casein kinase II phosphorylation sites. These sites may be associated with protein kinase phosphorylation and acylation, and site-directed mutagenesis experiments would confirm this. Positive selection site 234D was located in the functional domains of the 225–237 amino acids (MGTYFQVQDDYLD). This was tested in the branch-site model, which showed better than any other model that they had important and potential positive selection functions during evolution. Furthermore, positive-selection site 98S contained casein kinase II phosphorylation and chemical binding sites, such as Mg^2+^ binding site, which are relatively close in the space structure. It could be deduced that the 98S located in the highly conserved aspartate-rich region is the more important functional site. To further characterize the relationship between functional divergence and the site-specific evolution of amino acids, some potential amino acid sites associated with positive selection were chosen and mapped to the sequence alignment and the 3D structural model. The results showed that the functional divergence of the 98S site occurred during the site-specific evolution of amino acids, which suggested that 98S site-specific evolution was closely related to functional divergence in the FPS family.

## Conclusions

This study is the first large-scale evolutionary analysis of FPS in land plants. It explores the relationship between the molecular evolution of positive selection sites and their roles in plant FPS. Our results indicate: (1) FPS genes in plants appeared very early, and could be traced back to the bryophyte divergence to pteridophyte, which then evolved into gymnospermae, monocotyledonae, and dicotyledoneae; and (2) a number of signals for positive selection exist in plant FPSs. Thirty-nine positively selected sites in the site model and three positively selected sites in the branch-site model were detected, respectively. Furthermore, 98S was detected by both models and was located in the catalytic center. Therefore, 98S was considered the most significant site for plant FPS during the terpenoid synthesis process. 234D, which was detected in the branch-site model and was located in the functional domains, may provide an important reference for exploring further functional sites for FPS in the triterpenoid biosynthesis pathway. (3) The diversification of FPS genes among terrestrial plants could be attributed to functional divergence, which probably improves the activity of the enzymes in the triterpenoid biosynthesis pathway when plants adapt to terrestrial environments. This study provides useful information for further research on the regulation of triterpenoid biosynthesis.

## Methods

### Sequence data

In our study, plant FPS gene sequences contain two parts. FPS sequences in *Panax notoginseng* (GenBank accession AAY53905) and *Gynostemma pentaphyllum* (GenBank accession KJ917160) were cloned by our laboratory using rapid-amplification of cDNA ends (RACE) technology, and other cDNA sequences for FPS genes were collected from existing databases. The amino acid sequences were downloaded from GenBank at the National Center for Biotechnology Information (NCBI) (http://www.ncbi.nlm.nih.gov/) and the UniProt databases (http://www.uniprot.org/) (information about the total FPS sequences is shown in Additional file 1, downloaded before 2015-06). Then, BLAST and PSI-BLAST searches against the non-redundant database of FPS genomes at UniProt and NCBI were conducted. Only the full-length coding sequences were utilized in the final analysis. All partial, putative, redundant, and incomplete CDs were eliminated from our original sequences. In addition, each corresponding protein was matched to CDs. The final data included 68 sequences from terrestrial plants. These consisted of 2 gymnospermae, 10 monocotyledons, 54 dicotyledons, and *Chlamydomonas reinhardtii (CrFPS) and Huperzia serrate (HsFPS)* as outgroups.

### Sequence alignment

Multiple sequence alignments were performed using MUSCLE software [46] with the default parameters (http://www.ebi.ac.uk/Tools/msa/muscle/) to align the sequences of the proteins after the exclusion of poorly aligned positions, gap positions, and highly divergent regions. Then the CDs sequences were rearranged according to their amino acid alignment. The aligned amino acids and rearranged CDs were entered into EMBL web tool PAL2NAL [47] (http://www.bork.embl.de/pal2nal/), which can form multiple codon alignments from matching amino acid sequences. The nucleotide sequences after PAL2NAL alignment were then converted to the nexus format using MEGA4.0 software [48] for phylogenetic analysis.

### Phylogenetic analysis

Phylogenetic trees were generated using MrBayes version 3.1.2 software [49, 50]. Before the MrBayes tree could be constructed, we had to modify the parameters in the nexus file using PAUP* version 4.0 [51] and Modeltest version 3.7 [52] to produce test outfiles that could be used to obtain a list of the best settings for these parameter types. The Akaike Information Criterion (AIC) [53] in PAUP* version 4.0 was used to evaluate the estimate of the most appropriate model for amino acid substitution during the tree-building analysis. ML [54] optimizations and distance methods were evaluated by the PhyML program in PAUP* version 4.0. Then the likelihood settings were obtained from the best-fit model (GTR + I + G) selected by AIC [55] in Modeltest 3.7. It comprises three important commands that can be used to specify the evolutionary model (lset), the prior knowledge (prset), the generation time, and the sampling frequency (mcmc). The parameters added and modified in the nexus file for tree reconstruction were as follows: Statefreqpr = dirichlet (0.2722, 0.2343, 0.2413, and 0.2522), revmatpr = dirichlet (1.4781, 3.1597, 1.1667, 1.1255, 4.6277, and 1.0000), shapepr = fixed (2.2202), pinvarpr = fixed (0.0054), unlinkshape = (3), mcmcp ngen = 10,000,000, and samplefreq = 10,000; mcmc. There were 10 million generations with sampling every 10 thousand generations [56, 57]. After completing the MrBayes analysis, the first 250,000 generations were discarded from every run. The remaining data were used to compute the phylogenetic trees and to determine the posterior probabilities at the different nodes. When all the parameters had been completely modified, we used MrBayes to construct the phylogenetic tree [50].

### Detection of recombination events

According to previous research, LRT can lead to the false detection of positive selection in the presence of a recombination event [58]. Although recombination between species may occur in animals and plants, the sequence divergence is generally too low for phylogeny-based likelihood methods to be useful [59]. Recombination events may affect the detection of the positively-selective evidence. Therefore, we first tested for recombination signals between sequences involved in the alignment of FPS genes. The GARD approach [33] was applied to screen multiple sequence alignments for evidence of phylogenetic incongruence, and to identify the number and location of breakpoints and sequences involved in putative recombination events [34]. RDP software was also used to detect recombination events in FPS.

### Positively-selected sites and putative biological significance

To explore the selection pressure, we performed a strict statistical analysis using the CodeML program in the PAML version 4 software [60] using branch model, site model, and branch-site model [61] in a run based on the non-synonymous (dN) and synonymous (dS) nucleotide substitution rate ratio (dN/dS) or ω. Four files needed to be entered into CodeML: the nuc file, the treeview file, the corresponding ctl file, and the CodeML application program. The nuc file was produced from a DAMBE format conversion using PAML. If ω > 1, then there was a positive selection on some branches or sites, but the positive selection sites may occur in very short episodes or on only a few sites during the evolution of duplicated genes; ω < 1 suggests a purifying selection (selective constraints); and ω = 1 indicates neutral evolution. The parameter estimates (ω) and likelihood scores [62] were calculated for three pairs of models. These were M0 (one-ratio) vs. M3 (discrete), M1a (nearly-neutral) vs. M2a (positive-selection), and M7 (beta) vs. M8 (beta&ω) [50]. In these models, M0 assumed a constant ω ratio for all FPS coding sites; M3 allowed for three discrete classes of ω within the gene that was contrasted with LRT against the M0 model where the ω ratio was averaged over all gene sites; and M1a allowed for two classes of ω sites: negative sites with ω_0_ < 1 estimated from our data; and neutral sites with ω_1_ = 1, whereas M2a added a third class with ω_2_ possibly >1 estimated from our data. M7 was a null model in which ω was assumed to be beta-distributed among sites and M8 was an alternative selection model that allowed an extra category of positively selected sites [63]. The LRT [64] was used to compare the fit to the data of two nested models, which measured the statistical significance of each pair of nested models. The twice the log likelihood difference between each pair models (2ΔL) follows a Chi square distribution with the number of degrees of freedom equal to the difference in the number of free parameters. Therefore, we can get a p value for this LRT [65]. A significantly higher likelihood of the alterative model compared to the null model suggests positive selection. Generally, all positive selection sites were calculated by the M8 model, which provided some useful information for the branch-specific and branch-sites analysis. These site models might not detect positive selection affecting only a few sites along a few lineages after a duplication event, so we also implemented the branch model to select the statistically significant “foreground branch” under positive selection. This was achieved by comparing the fit to the data of the “one-ratio” model (M0) with the “free ratios” model (FR), where the rate parameters were estimated independently in each lineage. All other branches in the tree were “background” branches. The background branches share the same distribution of ω values among sites, whereas different values can apply to the foreground branch. Then, the branch-site model were applied, which further estimated the different dN/dS values among the significant branches detected by the branch model and among sites [66]. Finally, a Bayes empirical Bayes (BEB) [64] approach was then used to calculate the posterior probabilities that a site comes from the site class with ω > 1, which, when implemented in PAML4, were used to identify sites under positive selection or purifying selection in the foreground group with significant LRTs [64]. Each branch group was labeled as a foreground group as well.

### Positive selections in the protein sequences and structure analysis

All the protein sequences for FPS were aligned with Clustal W and displayed through GeneDoc (http://www.nrbsc.org/gfx/genedoc), which enabled us to examine the possible mechanisms driving the structural evolution of the FPS family in the triterpenoid biosynthesis pathway. First, the functional areas in the model are composed of positively-selected sites and post-translational modification sites, such as the highly conserved aspartate rich region located between positions 100 and 104 (DDSKD), protein kinase C phosphorylation sites, casein kinase II phosphorylation sites, N-myristoylation sites, amidation sites, and the conservative catalytic sites for isopentenyl pyrophosphate synthase (Trans-IPPS). Secondly, the positive selection sites related to the above functional sites were marked according to the experimental results. Thirdly, the transmembrane predictions and the post-translational modification sites above were determined with predict protein [67, 68] and TMHMM2.0 [69]. An estimate for the prediction accuracy was based on the confidence score for the modeling. Finally, PYMOL software version 1.5 [70] (http://www.pymol.org/) was used to predict the potential impact that those positive selected sites may have on the overall structure and function of the protein. Furthermore, the functional areas and relevant positive-selection sites identified in the evolutionary analysis were built into three dimensional graphic models and are shown by the highlighted parts.

## Additional file

**Additional file 1.** Appendix 1 FPS sequences information.

## Abbreviations

FPS: farnesyl pyrophosphate synthase
SS: squalene synthase
ML: maximum-likelihood
GARD: Genetic Algorithm for Recombination Detection
RDP: Recombination Detection Program
PP: posterior probability
RACE: rapid amplification of cDNA ends
BEB: Bayes empirical Bayes
LRT: likelihood ratio test
dN: non-synonymous
dS: synonymous
AIC: Akaike Information Criterion
